# Impact of ketamine on postoperative delirium: insights from trial sequential analysis

**DOI:** 10.1097/JS9.0000000000001010

**Published:** 2023-12-18

**Authors:** Kuo-Chuan Hung, I-Wen Chen, Ping-Hsin Liu

**Affiliations:** a Department of Anesthesiology, Chi Mei Medical Center; b Department of Anesthesiology, Chi Mei Medical Center, Liouying, Tainan City; c Department of Anesthesiology, E-Da Dachang Hospital, I-Shou University, Kaohsiung City, Taiwan

*Dear Editor,*

We read with great interest the systematic review by Fellous *et al*.^1^ on perioperative ketamine for the prevention of postoperative delirium and neurocognitive disorders after analyzing 1618 patients across 14 randomized controlled trials. The pathophysiological rationale for the benefits of ketamine is its modulation of neuroinflammation and anti-excitotoxic properties^2,3^, which are implicated in postoperative cognitive dysfunction. While the authors did not find a statistically significant difference in the primary outcomes of postoperative delirium or postoperative neurocognitive disorders with perioperative ketamine administration, the evidence may still be inadequate to draw definitive conclusions.

To address the issue, further analysis may be required. Trial sequential analysis (TSA) is a useful statistical technique to assess cumulative evidence in meta-analyses, accounting for repeated testing on accumulating data and the risk of random errors^4,5^. TSA estimates the required information size (number of participants) to reliably detect or reject an intervention effect. This helps to determine whether a meta-analysis has adequate power or if additional trials are needed. In addition, TSA can determine if there is firm evidence of futility that an intervention is unlikely to show benefit even if more trials are conducted. This can justify the early cessation of further studies on ineffective treatment.

As TSA was not performed in the original meta-analysis^1^, we conducted TSA on the raw data of the originally published meta-analysis to further evaluate the evidence. TSA was conducted using TSA viewer version 0.9.5.10 Beta (www.ctu.dk/tsa) as previously reported^4^. Statistical precision was established by setting type 1 error at 5%, the desired statistical power at 80%, and the magnitude of the intervention effect was detected as a 20% relative risk reduction. As shown in Figure 1, TSA demonstrated that the cumulative *z*-curve for postoperative delirium did not cross the required information size boundary or reach the futility boundary. The required information size to detect or reject a 20% relative risk reduction for postoperative delirium was ~6494 patients. This suggests that additional studies are needed to confirm or exclude an effect size of 20% before drawing definitive conclusions from the currently available evidence.

**Figure 1:**
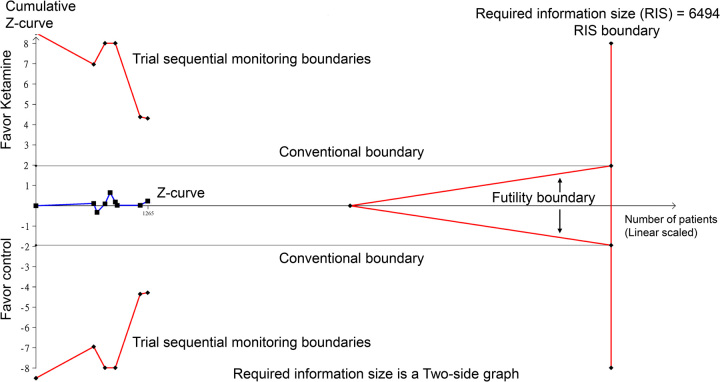
Trial sequential analysis (TSA) of the effect of perioperative ketamine on postoperative delirium. The required information size of 6494 patients was calculated based on a 20% relative risk reduction, with a type I error of 5% and power of 80%. The cumulative *z*-curve (blue line) does not cross the trial sequential monitoring boundaries for benefit or harm, or reach the required information size, indicating inconclusive evidence for both outcomes. Further trials are needed to assess the effects of perioperative ketamine administration reliably.

Therefore, while the originally published meta-analysis^1^ did not show significant differences with perioperative ketamine for reducing postoperative cognitive complications, the lack of adequate power based on TSA prevents conclusive interpretation. Further large randomized trials focusing on high-risk patients (e.g. the elderly) are still warranted to determine whether ketamine has any protective cognitive effects in surgical patients. We commend the authors for highlighting this clinical uncertainty, which will hopefully stimulate further research on optimizing postoperative cognitive outcomes.

## Ethical approval

Not applicable.

## Consent

Not applicable.

## Sources of funding

None.

## Author contribution

K.-C.H. and P.-H.L.: wrote the main manuscript text and I-W.C.: prepared Figure 1. All authors read and approved the final version of the manuscript.

## Conflicts of interest disclosure

The authors declare no conflicts of interest.

## Research registration unique identifying number (UIN)

Not applicable.

## Guarantor

Kuo-Chuan Hung.

## Data availability statement

The datasets used and/or analyzed in the current study are available from the corresponding author upon reasonable request.

## Provenance and peer review

This paper was not invited.
